# Notes on Microscopy

**Published:** 1875-09

**Authors:** Geo. E. Blackham


### MEDICAL NOTES.

ART. I.—*Notes on Microscopy.* By GEO. E. BLACKHAM, M. D.

I. CONJOINED EPITHELIUM.—Prof. Martin, of Bristol (England) Medical School has an interesting paper on the subject in the MICROSCOPICAL JOURNAL for August in which he discusses the peculiar form of epithelial cell discovered by Schultze some twelve years ago and called by him *Stachel und Riff Zellen* (prickle and ridge cells). These cells are covered or surrounded with spinous process, which appear to interlock with those of adjacent cells as the bristles of two brushes pressed together, or, as described by Mr. Stirling, of Edinburgh, like watch wheels. Prof. Martin combats the idea that these are really processes thrown out by the cells and terminating in free points or ends, and holds that they are in reality, continuous bands of formed material extending from cell to cell and the result of imperfect division of the formed material of the parent cells from which those thus united have been formed by fissaparous miltiplication.

II. Microscopical Examinations of the Process of Healing Ulcers by Transplantation of Skin.—M. Thiersch, having a patient whose leg had to be amputated in consequence of a large and incurable ulcer, thought it a good opportunity to examine the changes that took place when portions of skin are implanted on granulating surfaces. For three weeks previously, he accordingly transplanted portions of the skin day by day, the last being applied eighteen hours before amputation. The chief results were: 1. That adhesion occurs without the intervention of any immediate cementing substance The adherent parts are in immediate application or are, at most, only separated by a couple of blood corpuscles. 2. The adhesion, when complete, takes place by means of the inosculation of vessels, which may be observed even eighteen hours after the act of transplantation of new skin. A connection is, at this period seen to occur by intercellular passages extending between the sharply contoured vessels of the skin on one hand, and those of the granulations on the other, and these intercellular passages become proper vessels in the course of a few days. 3. At the same time, the vessels of the skin beneath the transplanted portion undergo secondary changes; they become wide, irregularly dilated, with prominences on their walls, and in fact assume the characters of embryonal blood vessels. 4. True, new formation of vessels may take place when the primary inosculation fails. In such cases, the epidermis and the papillary bodies fall off after a little while, and the transplantation is believed to have failed; but this is not so, since the subcutaneous connective tissue with the remains of the sweat glands remains adherent.

After the lapse of some time new formed epidermis appears where the transplantation was made, which may perhaps be due to the germination of the remains of the sweat glands. Thiersch finally recommends a modification of Rererdin's plan, viz: that the surface of the wound to which the skin is about to be transplanted shonld have any granulations that may be be found shaved off, and the new skin applied in the course of a few hours.—LONDON LANCET.

III. BIOLOGICAL AND MICROSCOPICAL SECTION OF THE ACADEMY OF NATURAL SCIENCES, PHILADELPHIA.—If there is anything in a name the proceedings of a body with so august a title as the above should be regarded with the deepest respect and the reports of their committees should be models of clear statements and wise conclusions. The report made in February last by Dr. J. Cheston Morris, "chairman of the committe appointed to examine optically the $\frac{1}{25}$ and $\frac{1}{50}$ objectives displayed at a late exhibition of the Section" does not, we regret to say, fulfill these indications as the following extract will show. The committee say: "The dependence of resolving power of upon angle of aperture is very well shown by placing a pleurosigma angulatum, for instance under a one fourth or *one tenth* with such an eyepiece as will amplify sufciently, and *putting the compound body horizontally in front of a direct light. In this position no lines will be seen, but by rotating the compound body on its axis an oblique light is obtained, which at different angles according to the power of the objective, will bring out transverse or oblique lines, and finally dots appearing as hexagons.*" Then follows a table showing the angles at which the various markings appeared with different objectives. The committee concluded their report as follows: "From the observations noted above we deduce one very important fact, viz: That the different appearances of lines, dots, hexagons, &c. on the pleurosigma angulatum are not only the varied results of angle of aperture of amplification and of illumination, *but that they may be obtained with less and less obliquity of light as we increase the power of the objective;* thus making it evident that high powers with direct central light show us clearly things which we rather guessed at than saw (owing to increased chance of spherical and chromatic aberration and distortion from the employment of oblique light) with lower ones. We would conclude therefore by recommending these high power lenses to those engaged in microscopic re_ search, &c., &c."

In the above quotations from the report the italics are the writer's, not the committee's, and are intended to render prominent certain points which are so remarkable as to demand notice. In the first place, with a microscope "arranged horizontally in front of a direct light," oblique light can *not* be obtained "by rotating *the compound body on its axis*," because such rotation, even were it possible (and very few first-class instruments in this country are so constructed), would not change the relation between the light and the object. The committee probably meant that oblique light could be obtained by turning the *entire stand* so as to make the tube point more or less away from the light. Next, with the microscope arranged as directed by the committee, the writer saw *with direct central light* the hexagons of the P. angulattum with an immersion of 1-10 of 160° made for him by Wm. Wales, of Fort Lee, N. J. The same result was obtained with the microscope in the upright position, and direct light reflected centrally from the plane mirror. The latter experiment was repeated with success with an immersion 1-10 of 135° without adjustment for cover recently sent me by Mr. Wales for examination, As the obliquity of light in these three experiments was *nil* and as the 1-10 showed with central light all there was to be seen, it does not appear that the results could have been *obtained with less and less obliquity of light by increasing the power to a* 1-25 or 1-50. The committee must have been unfortunate in their 1-10s or their manipulation, most probably the latter. No wonder that Dr. Hunt (a member of the committee) "wished it to be distinctly understood that he had nothing to do with the preparation of the report, and did not wish to be held responsible for the views advanced in it."

IV. Angle of Aperture.—The battle of the angles goes bravely on. Mr. Henry J. Slack, F. G. S., Sec'y R. M. S., has a long article in the London *Monthly Microscopical Journal* for May on "Angle of Aperture in Relation to Surface Markings and Accurate Vision," in which he takes strong ground in favor of small angles, and speaks with somewhat effusive enthusiasm of certain German objectives made by Zeiss of Jena, a C ($=\frac{1}{4}$) of 48° and a D (=1-6 of 72°, which resolved the Pleurosigma Hippocampus with direct light from a clear sky, using a B eyepiece with the former and an A eyepiece with the latter. The advantages he claims for Zeiss' low angle lenses are, greater working distance and ease of manipulation and a resolving power equal to that of higher angled English lenses. In the August number of the same journal are letters from Mr. Henry Mayall, Jr, and Mr. Jabez Hogg, sharply criticising Mr. Slack's positions both as to fact and to theory, and Mr. A. F. Dod, of Memphis, claims that on comparison with low angled glasses by the best American, English, French and German makers, a Tolles' immersion 1-10 of 180° (?), (the highest possible angle,) beat the low angled lenses on their own ground. besides doing many things which they failed to do in the way of resolution.

